# Management and Outcome After Early Renal Transplant Vein Thrombosis: A French Multicentre Observational Study of Real-Life Practice Over 24 Years

**DOI:** 10.3389/ti.2023.10556

**Published:** 2023-03-23

**Authors:** Ludivine Cambou, Clémentine Millet, Nicolas Terrier, Paolo Malvezzi, Marc-Olivier Timsit, Dany Anglicheau, Lionel Badet, Emmanuel Morelon, Thomas Prudhomme, Nassim Kamar, Anne Lejay, Peggy Perrin, Charlotte Uro-Coste, Bruno Pereira, Anne Elisabeth Heng, Cyril Garrouste, Laurent Guy

**Affiliations:** ^1^ Clermont-Ferrand University Hospital, Department of Urology, Clermont-Ferrand, France; ^2^ Service d’Urologie, CHU Grenoble-Alpes, Grenoble, France; ^3^ Service de Néphrologie, Hémodialyse, Aphérèses et Transplantation Rénale, CHU Grenoble-Alpes, Grenoble, France; ^4^ Necker Hospital, Assistance Publique-Hôpitaux de Paris, Department of Urology, Paris, France; ^5^ Necker Hospital, Assistance Publique-Hôpitaux de Paris, Department of Nephrology and Kidney Transplantation, Paris, France; ^6^ Service d’Urologie, Hôpital Edouard Herriot, Hospices Civils de Lyon, Lyon, France; ^7^ Service de Néphrologie, Hôpital Edouard Herriot, Hospices Civils de Lyon, Lyon, France; ^8^ Toulouse University Hospital, Department of Urology, Toulouse, France; ^9^ Toulouse University Hospital, Department of Nephrology, Toulouse, France; ^10^ Department of Vascular Surgery and Kidney Transplantation, University of Strasbourg, Strasbourg, France; ^11^ Department of Nephrology and Transplantation, University Hospital, Strasbourg, France; ^12^ Fédération de Médecine Translationnelle (FMTS), Strasbourg, France; ^13^ INSERM U1109, LabEx TRANSPLANTEX, Strasbourg, France; ^14^ Clermont-Ferrand University Hospital, Department of Nephrology, 3iHP, Clermont-Ferrand, France; ^15^ Clermont-Ferrand University Hospital, Biostatistics Unit (DRCI), Clermont-Ferrand, France

**Keywords:** kidney transplant, vein thrombosis, early vein thrombosis, outcome, management

## Abstract

Early (<14 days) renal transplant vein thrombosis posttransplant (eRVTPT) is a rare but threatening complication. We aimed to assess eRVTPT management and the rate of functional renal transplantation. Of 11,172 adult patients who had undergone transplantation between 01/1997 and 12/2020 at 6 French centres, we identified 176 patients with eRVTPT (1.6%): 16 intraoperative (Group 1, G1) and 160 postoperative (Group 2, G2). All but one patient received surgical management. Patients in group G2 had at least one imaging test for diagnostic confirmation (N = 157, 98%). During the operative management of the G2 group, transplantectomy for graft necrosis was performed immediately in 59.1% of cases. In both groups, either of two techniques was preferred, namely, thrombectomy by renal venotomy or thrombectomy + venous anastomosis repair, with no difference in the functional graft rate (FGR) at hospital discharge (*p* = NS). The FGR was 62.5% in G1 and 8.1% in G2 (*p* < 0.001). Numerous complications occurred during the initial hospitalization: 38 patients had a postoperative infection (21.6%), 5 experienced haemorrhagic shock (2.8%), 29 exhibited a haematoma (16.5%), and 97 (55.1%) received a blood transfusion. Five patients died (2.8%). Our study confirms the very poor prognosis of early renal graft venous thrombosis.

## Graphical Abstract

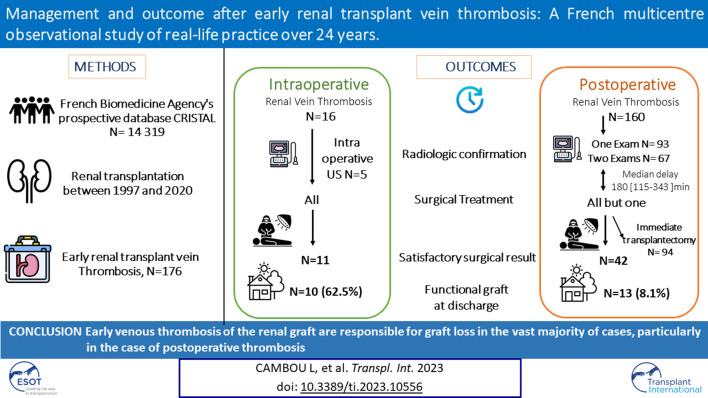

## Introduction

Early renal vein thrombosis posttransplantation (eRVTPT) is a serious complication occurring during the first 14 days of renal transplantation (1), and its frequency is estimated to be between 0.1% and 5.5% (2–6). It is very often accompanied not only by graft loss (7) due to an absence of collaterality with venous flow coming only from the renal vein of the transplant (8) but also by embolic and/or haemorrhagic complications that can lead to death. eRVTPT should be suspected in the presence of pain that is not relieved by the usual analgesic treatments, the occurrence of oligoanuria, an excessively productive drainage or even an increase in macroscopic haematuria and a deterioration of renal function (2,7). Clinical suspicion can be confirmed by renal Doppler ultrasound (9), computed tomography angiography, or magnetic resonance angiography (10,11).

To date, there is no recommendation concerning the management of eRVTPT. Indeed, the data on such management in the literature are based on case series or small cohorts. It is reported to require surgical revision, with thrombectomy by renal venotomy (12), anastomotic repair, or explantation, flushing with preservative solution and reimplantation (13), and more rarely endovascular treatment (14) or thrombolysis alone (15). Regardless of the reported management, the rate of functional grafts at discharge is extremely low (5–7,16).

The aim of our study was to investigate different management strategies during the occurrence of eRVTPT and the outcome of the renal graft.

## Patients and Methods

### Patients and General Data

This retrospective multicentre observational study was conducted at 6 French adult renal transplantation centres: Gabriel Montpied Hospital, Clermont-Ferrand University Hospital; Michallon Hospital, Grenoble University Hospital; Necker-Enfants Malades Hospital, AP-HP; Edouard Herriot Hospital, Lyon University Hospital; Rangueil Hospital, Toulouse University Hospital; and Nouvel Hospital Civil, Strasbourg University Hospital.

The inclusion criteria were patients aged more than 18 years who had undergone renal transplantation between 01/01/1997 and 31/12/2020 complicated by venous thrombosis of the graft during the initial hospitalization (<14 days). To avoid selection bias, we submitted a request to the Biomedicine Agency database with the following terms: “vascular complications” and/or “no primary function.” We then checked all medical records and included only patients with early vein thrombosis of the allograft.

We collected the following demographic and clinical characteristics of the donor from the Biomedicine Agency’s prospective database CRISTAL and possibly from the patient’s file: type of donor (living or deceased), age, so-called “marginal” donor with extensive selection criteria (17), presence of thromboembolic risk factors, year of transplantation, laterality of the kidney, possible anatomical abnormalities, and conditions of retrieval. We also collected the following demographic, clinical and biological data of the recipient: age, sex, body mass index, thromboembolic history, haematological pathologies, history of miscarriage, smoking, diabetes, initial renal disease, presence of pretransplant anticoagulant or antiaggregant treatments, and induction immunosuppressive treatments.

Intraoperative graft data were collected from operative reports. We distinguished the type of graft (bitransplant, multiorgan transplant, or renal transplant alone) and the duration of cold and warm ischaemia; from the operative reports, we identified any difficulties that occurred during vascular anastomoses and the flushing or non-flushing of the vessels intraoperatively. We also noted signs suggestive of renal graft vein thrombosis, whether clinical and/or biological, and imaging studies allowing us to confirm this, as well as the management, the functional results in the long term, and the complications secondary to this management.

### Definition of Groups

**FIGURE 1:**
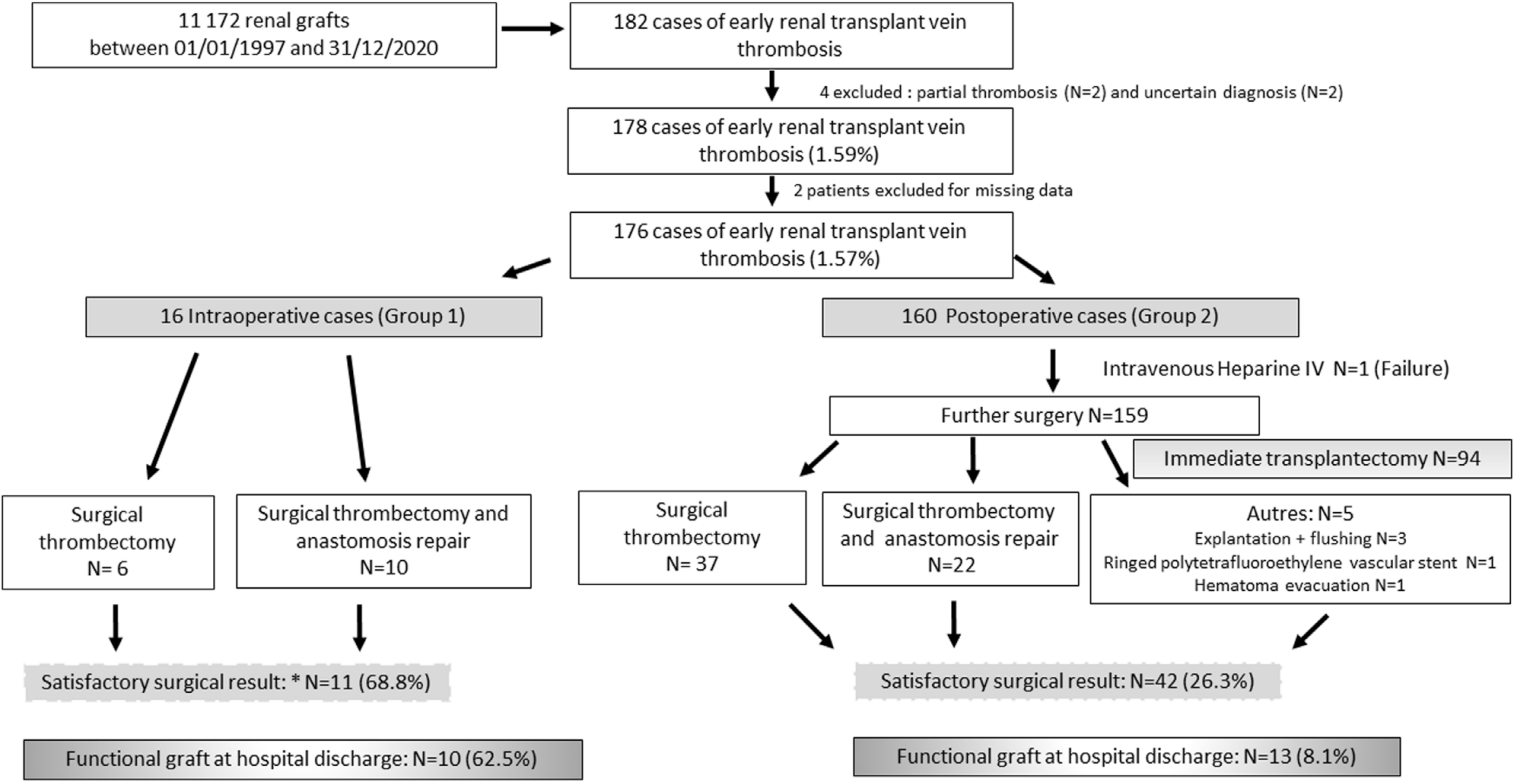
Flow Chart.

Of the 14,319 renal transplants (RTs) performed at these 6 centres in the period from 01/01/1997 to 12/31/2020, 182 (1.3%) patients presented with renal graft vein thrombosis during the initial transplant hospitalization. We excluded 2 patients with partial thromboses, 2 patients for whom the diagnosis was uncertain, and 2 patients because of a lack of data (Figure 1).

Patients were divided into 2 groups. Group 1 (G1; N = 16) included patients with intraoperative renal vein thrombosis. Group 2 (G2; N = 160) included patients with a postoperative diagnosis of eRVTPT.

### Statistical Analysis

Statistical analysis was essentially exploratory to describe management strategies in the event of eRVTPT. Data are presented as the mean and standard deviation or the median and interquartile range. The assumption of normality distribution was studied with the Shapiro–Wilk test. The chi-square test or, if appropriate, Fisher’s exact test was used to compare independent groups concerning graft outcomes, such as the proportion of patients with a functional graft rate at hospital discharge or the time to thrombosis. Statistical analysis was performed using the Stata software (version 15, StataCorp, College Station, Texas, US). The statistical tests were two-sided, with type I error set at 0.05. We performed a study of the factors associated with functional grafts at discharge using generalized linear mixed models with a logit link function to model between- and within-centre variability (as random effects). For multivariate analyses, we performed a multiple mixed logistic regression that considered covariables in terms of their significant results in univariate analysis (*p* < 0.10) (Table 1) as well as their clinical relevance (2,18–20). The results are expressed in terms of odds ratios (ORs) and 95% confidence intervals (95% CIs).

**TABLE 1:** Characteristics of the kidney transplant recipients (N = 176).

	All (N = 176)	G1 (N = 16)	G2 (=160)	*p*
Cardiovascular risk factors				
Age	56.5 [45.5–65.0]	61.5 [45–70.5]	55.0 [45.5–64.5]	0.33
Male	99 (56.2)	6 (37.5)	93 (58.1)	0.11
BMI at transplant. kg/m^2^	25.8 [22.6–29.3]	28.7 [25.5–31.4]	25.7 [22.4–29.2]	**0.03**
Diabetes	31 (17.6)	5 (31.2)	26 (16.3)	0.30
Smoking	29 (16.4)	2 (13.3)	27 (16.7)	0.67
Risk factors for venous thrombosis				
History of venous thrombosis	34 (19.3)	6 (37.5)	28 (17.5)	**0.05**
Vein thrombosis on a previous graft	3 (1.7)	0	3 (1.8)	0.52
Arteriovenous fistula thrombosis	15 (8.5)	3 (18.7)	12 (7.5)	0.12
Deep vein thrombosis and/or pulmonary embolism	34 (19.3)	6 (37.5)	28 (17.5)	0.05
Central line thrombosis	1 (0.6)	0	1 (0.6)	1.00
Systemic pathologies	16 (9.1)	1 (6.3)	15 (9.3)	1.00
Systemic autoimmune diseases	2 (1.1)	0	2 (1.3)	
Coagulation disorders	9 (5.1)	1 (6.3)	8 (5)	
Haematological pathologies	5 (2.8)	0	5 (3.1)	
Surgery within previous 3 months	6 (3.6)	3 (18.7)	3 (1.8)	**0.01**
Initial Nephropathy				0.18
Glomerulopathy	53 (30.1)	4 (25.0)	48 (27.7)	
Vascular nephropathy	25 (14.2)	6 (37.5)	19 (10.7)	
Polycystic kidney disease	23 (13.1)	2 (12.5)	21 (13.1)	
Diabetic nephropathy	21 (11.9)	3 (18.7)	18 (11.2)	
Unknown	18 (10.2)	0	18 (11.2)	
Malformative uropathies	14 (7.9)	0	14 (7.9)	
Chronic Interstitial Nephropathy	14 (7.9)	1 (6.25)	13 (7.3)	
Vasculitis/Connectivities	8 (4.6)	0	8 5)	
Usual treatment at Transplantation Day				
Anticoagulants	13 (7.3)	0	13 (8.1)	0.23
Antiaggregants	38 (21.6)	4 (25)	34 (21.2)	0.72
Induction immunosuppressive regimen	N = 146	N = 16	N = 130	
Thymoglobulin	91 (62.3)	10 (62.5)	81 (62.3)	0.71
Basiliximab	51 (34.9)	5 (31.2)	46 (32.3)	0.89
Unknown	4 (2.7)	1 (6.2)	3 (2.3)	

Data are presented as the number of patients (associated percentages), as the mean ± standard deviation, or as the median [interquartile range]. Bold values denote statistical significance at the p< 0.05 level.

Abbreviations: BMI, body mass index; G1, Group 1 included patients with intraoperative renal vein thrombosis; G2, Group 2 included patients with a postoperative diagnosis of eRVTPT.

## Results

### Characteristics of Patients With Early RT Vein Thrombosis

The characteristics of the patients are given in Table 1. The 176 patients included in our study were predominantly male (56.2%), aged 56.5 ± 10.0 years at transplantation, and had a mean body mass index of 26.2 ± 3 kg/m^2^. In this cohort, thirty-one patients were diabetic (17.2%), and 16 (9.5%) were active smokers. A history of thrombosis was reported in 34 patients (19.0%), 12 of whom had multiple thromboses (6.8%). Haematological, haemostasis or immunological pathology posing a risk of thrombosis was found in 9.1% of cases (N = 16). Thirty-eight patients (21.6%) were receiving antiaggregation therapy, and 13 (7.3%) were receiving anticoagulant therapy (Table 1).

In the vast majority of cases, patients received a first kidney transplant (N = 144, 84.2%) from a deceased brain-dead donor (N = 166, 94.3%). The median donor age was 60 [46–71] years; 51.2% of the grafts (85/166) were considered marginal (Table 2).

**TABLE 2:** Transplantation characteristics (N = 176).

	All (N = 176)	G1 (N = 16)	G2 (N = 160)	*p*
Donors				
Age. median (IQR)	60 [46–71]	64.2 [48–78]	57.9 [45–70]	0.16
Deceased donor with expanded criteria	86/170 (50.6)	9/16 (56.2)	77/154 (50)	0.31
Living donor	10 (5.6)	0	10 (6.25)	0.60
Disseminated intravascular coagulation	2 (1.1)	0	2 (1.25)	0.65
Cold ischaemic time. min (N = 161)	918 [714–1259]	868 [780–1534]	919 [615–1255]	0.47
Difficult cannulation	4/155 (2.6)	2 (12.5)	2 (1.25)	**0.004**
Transplant				
First kidney transplantation	144/171 (84.2)	15 (93.7)	129 (83.2)	0.71
Single kidney	163 (93)	16 (100)	147 (91.8)	0.24
Dual kidney	8 4)	0	8 (4.5)	0.36
Multiorgan transplantation	5 3)	0	5 (3.13)	0.47
Right kidney	101/175 (57.7)	8 (50)	93 (58.4)	0.51
Immunology (N = 124)				
DSA	7 (5.6)	1/16 (6.3)	6/108 (5.6)	0.91
Panel-reactive antibody ≥85%	16 (12.9)	2/16 (12.5)	14/108 (12.9)	0.95
Intraoperative data				
Warm ischaemic time. min (N = 161)	45 [34–55]	47 [42–55]	475 [33–55]	0.60
Vein anatomy abnormality	23/172 (13.4)	1 (6.2)	22/156 (14.1)	0.58
Multiple veins with at least one sacrificed	11 (6.4)	0	11/156 (7.1)	
Vascular wounds at retrieval	10 (5.8)	0	9/156 (5.7)	
Fibromuscular dysplasia	2 (1.2)	1 (6.2)	1/156 (0.6)	
Venous anastomosis revision	19/159 (11.9)	9/16 (56.2)	10/143 (6.9)	**<0.001**
Renal vein twist	1 (0.6)	0	1/143 (0.7)	
Partial thrombus	2 (1.3)	0	2/143 (1.4)	
Strangulation of the iliac vein	1 (0.6)	0	1/143 (0.7)	
Not specified	3 (1.9)	0	3/143 (2.1)	
Arterial anatomy abnormality	55/170 (32.3)	4/16 (25.0)	51/144 (35.4)	0.41
Multiple arteries	37 (2.8)	0	37/144 (25.7)	
Atherosclerotic plaque	14 (8.2)	2/16 (12.5)	12/144 (8.3)	
Vascular wounds at retrieval	2 (1.2)	2/16 (12.5)	0	
Not specified	2 (1.2)	0	2/144 (1.3)	
Intraoperative heparin therapy	13/138 (9.4)	4/14 (28.5)	9/124 (7.3)	**0.01**
Vessel flushing	37/176 (21.0)	4/16 (25.0)	33/160 (20.6)	0.63

Data are presented as the number of patients (associated percentages). as the mean ± standard deviation. or as the median [interquartile range]. Bold values denote statistical significance at the p< 0.05 level.

DSA, donor-specific antigen; G1, Group 1 included patients with intraoperative renal vein thrombosis; G2, Group 2 included patients with a postoperative diagnosis of eRVTPT.

Among the patients who received a kidney transplant from a living donor, 1 received a right kidney, 3 others received a left kidney with a short and thin vein and 1 with 2 veins that had been ligated. eRVTPT was more frequent in patients who received a right kidney than in those who received a left kidney during the interest period (101/6421 (1.57%) patients vs. 74/7797 (0.95%) patients, *p* = 0.002).

### Initial Renal Transplantation Surgery

The median cold ischaemia time was 918 [714–1259] minutes. The median warm ischaemia time to perform vascular anastomosis was 45 [34–55] minutes. In 23 recipients (13.4%), the venous anatomy of the graft was abnormal (11 multiple veins, 10 venous wounds at harvesting, and 2 dysplastic veins). In addition, seven operators had to redo the venous anastomosis during the transplantation procedure (4.4%). During the initial operative procedure, 37 patients received a flush solution with heparinized saline or normal saline (27.4%), and 13 patients (9.4%) received heparin therapy (Table 2).

### Diagnosis of Intraoperative RT Vein Thrombosis (Group 1)

Half of the diagnoses of intraoperative RT vein thrombosis were made at one centre. All patients with intraoperative eRVTPT at this centre had a functional graft at discharge. This team frequently used intraoperative ultrasound during kidney transplant procedures to assess graft vascular anastomoses and flow. Recently, another centre introduced this technique and diagnosed one case of intraoperative eRVTPT with a favourable outcome.

### Diagnosis of Early Postoperative RT Vein Thrombosis (Group 2)

In the majority of cases, venous thrombosis was symptomatic (86.2%). It manifested as oligoanuria (63.1%), abnormal pain in the renal pelvis (26.9%) and frank haematuria (17.5%). Venous thrombosis was revealed by haemodynamic disorders, such as the use of vasopressor amines in 14 patients (8.6%) or haemorrhagic shock in 7 other recipients (4.3%). The main biological criterion leading to the diagnosis was increased creatinine levels (81/160; 50.6%). The vast majority of patients underwent imaging to confirm graft vein thrombosis: 68/160 (42.5%) by graft Doppler ultrasound, 22 (13.8%) by abdominopelvic computed tomography angiography, 61 (38.1%) by both aforementioned modalities, and 6 (3.7%) by ultrasound combined with MRI (Table 3).

**TABLE 3:** Postoperative diagnosis of graft vein thrombosis (Group 2. N = 160).

Time from transplant to diagnosis. hours	48 [24–120]
Clinical signs suggestive of venous thrombosis	
Asymptomatic	22 (13.8)
Oligoanuria	101 (63.1)
Abnormal pain	43 (26.9)
Macroscopic haematuria	28 (17.5)
Haemodynamic disorders	14 (8.6)
Haemorrhagic shock	7 (4.3)
Productive Redon catheter (blood)	6 (3.7)
Fever	3 (1.9)
Others (oedema. testicular pain)	3 (1.9)
Biological criteria suggestive of venous thrombosis	
None	37 (23.1)
Increased serum creatinine	81 (50.6)
Increased LDH levels	17 (10.6)
Hyperlactatemia	10 (6.3)
Thrombopenia	6 (3.7)
Anaemia	5 (3.1)
Inflammatory syndrome	3 (1.8)
Increased CK levels	2 (1.2)
Hyperkalaemia	2 (1.2)
Increased AST levels	1 (0.6)
Radiological examinations	
None	3 (1.9)
Doppler ultrasound	69 (42.5)
Computed tomography angiography	22 (13.8)
Doppler ultrasound and MRI	6 (3.8)
Doppler ultrasound + computed tomography angiography	61 (3.1)
Time between first radiological examination and second surgery. min (*n* = 75) 180 [115–342]	
1 exam (N = 41)	180 [106–300]
2 exams (N = 34)	215 [118–344]
Concomitant thrombosis. yes	44 (27.5)
Deep vein thrombosis	13 (8.1)
Thrombosis of the graft artery	13 (8.1)
Pulmonary embolism	10 (6.3)
Arteriovenous fistula thrombosis	8 (5.0)

Data are presented as the number of patients (associated percentages) or as the median [interquartile range].

AST, aspartate aminotransferase; CK, creatinine kinase; LDH, lactate dehydrogenase; MRI, magnetic resonance imaging.

### Therapeutic Management

In group 1, 10/16 (62.5%) intraoperative venous thromboses were treated surgically by repair of the venous anastomosis (Figure 1), and 6/16 (37.5%) were treated by venotomy for thrombectomy. Four (25.0%) patients underwent intraoperative transplantectomy, and 1 (6.3%) underwent secondary transplantectomy (Figure 1).

In group 2, the time to onset of venous thrombosis was 48 [24–120] hours (Table 3) after transplant surgery. Management was almost exclusively surgical (159/160, 99.4%), with the exception of one patient who received heparin therapy alone (Figure 1). In 94/159 patients (59.1%), transplantectomy was performed immediately because of a necrotic renal graft. For the other 65 patients (40.9%), the 2 main surgical techniques used were venotomy and thrombectomy (N = 37) or thrombectomy added to venous anastomosis repair (N = 22). For one patient, the type of surgical revision was not specified (Figure 1). Surgical revision was accompanied by primary procedure failure in 23/159 (14.5%) cases with intraoperative transplantectomy. For 42 patients (42/159; 26.4%), surgical revision was said to be satisfactory because of macroscopically satisfactory revascularization of the graft (Figure 1). However, 22/42 (52.4%) patients required a secondary transplantectomy during the initial stay, and only 13/42 (31.0%) had a functional graft vs. 10/11 (90.9%) in G1 (*p* = 0.001). Among the 7 patients discharged with a non-functional graft in place, 2 died in the weeks that followed, 3 benefited from a transplantectomy several months after the transplant for graft intolerance syndrome, 1 benefited from graft embolization, and the last patient kept the graft in place.

In 96 patients (62.8%), a pathology report of the allograft nephrectomies was available for analysis and confirmed the renal infarction due to renal thrombosis. None of the patients had signs of acute rejection.

### Complications of Early Postoperative RT Vein Thrombosis

The majority of patients in our cohort had significant blood loss defined as the need for at least one red blood cell transfusion (N = 131; 74.4%). Among them, 5 patients (2.8%) presented haemorrhagic shock, and 29 developed large haematomas (29/176; 16.5%). Thirty-one patients presented concomitant deep vein thrombosis and/or pulmonary embolism (Table 3). All received curative anticoagulant treatment that may contribute to significant blood loss. Thirty-eight patients presented a postoperative infection (Table 4).

**TABLE 4:** Complications associated with early RT vein thrombosis (N = 176).

	All (N = 176)	G1 (N = 16)	G2 (N=160)	*p*
Mortality	5 (2.8)	1 (6.2)	4 (2.5)	0.39
Intraoperative death during revision surgery	1 (0.6)	0	1 (0.6)	1.00
Between Day 0 and Day 15	3 (1.7)	1 (6.2)	2 (1.2)	0.25
Between Day 15 and Day 30	0	0	0	NA
Between Day 30 and Day 90	1 (0.6)	0	1 (0.6)	1.00
Complications				
Blood transfusion	97 (55.1)	9 (56.2)	88 (55)	0.92
Haematoma	29 (16.5)	1 (6.2)	28 (17.5)	0.48
Haemorrhagic shock	5 (2.8)	2 (12.4)	3 (1.8)	0.07
Postoperative infection	38 (21.6)	1 (6.2)	37 (23.1)	0.20
Urinary tract infection	11 (6.2)	0	11 (6.9)	0.60
Surgical site infection	9 (5.1)	1 (6.2)	8 (5.0)	0.59
Pneumonia	8 (4.5)	0	8 (5.0)	1.00

Data are presented as the number of patients (associated percentages) or as the median [interquartile range].

Five patients (5/176; 2.8%) died during initial management, including 4/176 (2.3%) within the first 15 days of transplantation. In G1, 1 patient died on day 4 from haemorrhagic shock. Four patients died in G2: 1 in the operating room from haemorrhagic shock during transplantectomy, 1 on day 6 from anaemia and hyperkalaemia, and the other 2 on day 11 and day 61 because of multivisceral failure, preceded by multiple repeat operations (Table 4).

### Patient Outcome After Hospital Discharge

Overall, the proportion of patients with a functional graft at discharge was 13.1% (N = 23). The proportion of patients with a functional graft at discharge from the hospital in the G1 group compared with the G2 group was 10/16 (62.5%) and 13/160 (8.1%), respectively (*p* < 0.001). A sensitivity analysis excluding patients who underwent dual kidney transplantation (N = 8) or multiorgan transplantation (N = 5) exhibited a similar rate of remission (data not shown). In multivariate analysis, patients who had a postoperative diagnosis of eRVTPT had a lower probability of having a functional graft at discharge (OR = 0.016, 95% CI [0.002; 0.119], *p* < 001).

All these grafts were also functional at 1 year. The median serum creatinine at 1 year was 155 [130–207] µmol/L, with similar values in the 2 groups (data not shown).

At 5 years, 16 patients had a functional graft, 2 patients were dialysed, 1 patient died, and 4 were lost to follow-up. In addition, 44/153 (29.1%) of the patients who lost their graft were able to receive a new transplant. None of the patients had thrombosis of their new graft (Table 4).

## Discussion

To our knowledge, this study is the largest series describing the management of eRVTPT (<14 days) and the first to describe the prognosis of intraoperative thrombosis (G1) and postoperative (G2) thrombosis. We reported an incidence of venous thrombosis of 1.4%, which is a rate comparable to that in the literature (6,16). In our cohort, venous thrombosis was responsible for graft loss in 86.4% of cases, a rate close to that in the literature (5,16). Only intraoperative thrombosis is associated with better graft survival (63.5%), which is probably due to the possibility of immediate management (13). Indeed, thrombosis of the RT vein is responsible for a decrease in blood flow at the microvascular level, resulting in renal ischaemia lesions. In the case of “surgical recovery,” the RT has undergone new ischaemia–reperfusion lesions with the consequences of a delay in the resumption of function due to tubular necrosis or even cortical necrosis, chronic dysfunction of the graft due to endothelial-mesenchymal transition and acute or chronic rejection lesions (21–23). Thus, at discharge, the graft was functional after “satisfactory” revascularization in 10/11 patients with intraoperative thrombosis (G1) and in 13 of 42 patients with postoperative thrombosis (G2) (*p* < 0.001). Therefore, a major challenge is to preserve or remove the kidney at the time of salvage surgery in G2 patients to avoid complications, i.e., haemorrhage or infection, or a new nephrectomy surgery. To help the surgical decision-making process related to this emergency surgery (24–26), further tools must be investigated. Ultrasound, a first-line imaging examination, in particular contrast-enhanced ultrasound (27,28), may be helpful in verifying macrovessel vascularisation but also parenchymal perfusion. Contrast-enhanced ultrasound can be easily performed intraoperatively to assist in decision making in case of doubt during initial surgery (G1) (27,29) or to assess viability (27,30) of the RT during rescue surgery but also at the bedside (28) to confirm the diagnosis. In our cohort, the diagnosis of intraoperative RT vein thrombosis was made by intraoperative ultrasound in 5 patients with favourable outcomes in all cases.

eRVTPT management was almost exclusively surgical. Indeed, in our cohort, only one patient was treated with curative-dose heparin therapy, without success. In the literature, only one case of curative dose heparin therapy with preservation of graft function has been reported (31) in a patient with late-onset venous graft thrombosis more than 9 years after transplantation. The use of heparin in our cohort was infrequent, both at the time of surgery (15.0%) and as a curative measure after surgery (6.3%). These data are probably explained by the fear of bleeding risk immediately posttransplantation (17), urging caution by medical and surgical teams. Exceptionally, other therapies have been reported in cases of early thrombosis, such as thrombolysis (15,32) or thromboaspiration followed by heparin therapy in curative doses (14). These techniques are most often proposed in cases of late venous thrombosis (33–35).

It should be noted that an immediate transplantectomy was performed in nearly 3 out of 5 cases when the graft was necrotic at the time of the revision surgery (G2). In the case of a viable graft, the two most common revascularization techniques were thrombectomy by venotomy and anastomotic repair. In the face of intraoperative venous thrombosis (G1), anastomotic repair is the most favoured technique. This is most often justified by a surgical imperfection at the origin of this thrombosis, requiring complete repair of the venous anastomosis: a twist in the vein, strangulation of the iliac vein, folding of the vein during positioning of the graft, folding over a long vein, a disparity in calibre between the vessels of the graft and those of the recipient, or external compression (3). The 2 complete explantations of the graft with flushing and reimplantation were not effective, contrary to the results reported in a retrospective series of 5 patients with venous complications. However, only 1 patient had vein thrombosis (36).

Morbidity remains high following the occurrence of venous thrombosis. Indeed, in our data collection, we observed 5 deaths, 4 of which occurred within the first 15 days of the transplant. The other complications observed were 5 cases of haemorrhagic shock, 38 (21.6%) postoperative infections, and a requirement for blood transfusion in more than half of the patients (55.1%). This may limit access to a new transplant due to immunization against the human leukocyte antigen system (37,38). However, 44 patients who lost their graft (29.1%) received a new kidney transplant after a median waiting time of 1 year. Indeed, the French Biomedicine Agency takes into account list seniority on the transplant waiting list in cases of early loss of graft function below 3 months.

Our work has several limitations. First, we report the results of a retrospective cohort. Thus, some difficulties during kidney retrieval or transplantation may have been overlooked. Second, at the time of revision surgery (G2), 94 (59.7%) transplantectomies were performed on a necrotic graft. There may have been a delay in diagnosis and/or management. Indeed, the clinical signs of venous thrombosis are aspecific (pain, oligoanuria, macroscopic haematuria) but must evoke the diagnosis (2). Serum LDH monitoring can aid in the diagnosis of thrombosis and should be measured daily during initial hospitalization (39). On the other hand, when the diagnosis was highly suspicious on ultrasound (40), 67/157 (42.7%) patients underwent another imaging procedure, which may have increased the delay in management. Therefore, the median time between the first radiological examination and salvage surgery was 180 [115–342] minutes (Table 3). In our study, 5/67 (7.5%) patients who benefited from two radiological examinations had a functional graft at discharge compared with 8/93 (8.6%) who had only one or none, *p* = NS. Third, we cannot exclude that some patients had abdominal compartment syndrome manifested by profuse bleeding (N = 13). All but one had immediate transplantectomy. The last patient underwent haematoma evacuation with a favourable outcome. In the case of suspected renal compartment syndrome, placing the graft intraperitoneally during salvage surgery may be proposed. Another limitation of our study is the absence of a control group, which prevents us from comparing medical (thrombophilia) and surgical aetiologies. Indeed, many of the following risk factors were identified (41): the occurrence of a perioperative haemodynamic disorder in the recipient, a history of thrombosis and/or diabetes in the recipient, and deceased donors aged less than 6 years or more than 60 years. This last factor remains controversial (42). In our series, grafts from marginal donors (18) represented approximately 51% of our cohort, which is comparable to the data from the French Biomedicine Agency (43). It has also been reported that there is an increased risk of thrombosis in the case of a right kidney, as in our study (44,45).

In conclusion, our study confirms the extreme severity of early venous thrombosis of the renal graft, which is responsible for graft loss in the vast majority of cases, particularly in the case of postoperative thrombosis. Although the prognosis is poor, its management is mostly surgical and relies on immediate intraoperative venotomy for thrombectomy or thrombectomy and anastomotic repair. Further studies should allow us to better identify patients at risk of venous thrombosis to ensure close monitoring and to facilitate the development of appropriate thromboprophylaxis protocols.

## Collaborators for this Study

Julien Aniort, Alba Atenza, Xavier Bisbard, Fanny Buron, Florian Ceruti, Cyril Charbonnel, Clarisse Greze, Thomas Jouve, Christophe Legendre, Stephan Levy, Xavier Matillon, Sophie Ohlmann, Carole Philipponnet, Anne Ravel, Lionel Rostaing, Frederico Salusto, Nicolas Vedrine, Clementine Nicolo.

## Data Availability

The data analyzed in this study is subject to the following licenses/restrictions: General Data Protection Regulation - CNIL- https://www.cnil.fr. Requests to access these datasets should be directed to the corresponding author.

## Abbreviations

AST, Aspartate aminotransferase; CK, Creatinine kinase; eRVTPT, Early (<14 days) renal transplant vein thrombosis posttransplant; FGR, Functional graft rate; LDH, Lactate dehydrogenase; MRI, Magnetic resonance imaging; RT, Renal transplant.
